# Supplementary material for: Skin Barrier Function and Infant Tidal Flow-Volume Loops—A Population-Based Observational Study
Source: Children (Basel). 2022 Dec 31;10(1):88. doi: 10.3390/children10010088 (PMC9856825; doi:10.3390/children10010088)
Supplement: Supplementary file 1 [file children-10-00088-s001.zip › children-2100800-supplementary.pdf]

Supplementary Table S1. Summary statistics of TFV loop parameters in the 899 included three-month-old infants, as well as infants with lower t_{PTEF}/t_E and t_{PTEF}.

Characteristics	Mean (SD)	Median (IQR; min-max)	Mean (SD)	Median (IQR; min-max)	Mean (SD)	Median (IQR; min-max)
	Infants with TFV loops (n=899)		Lower t_{PTEF}/t_E [a] (n=48)		Lower t_{PTEF} [a] (n=223)	
t_{PTEF}/t_E	0.39 (0.08)	0.39 (0.12; 0.19-0.63)	0.23 (0.02)	0.24 (0.02; 0.19-0.24)	0.34 (0.08)	0.34 (0.11; 0.19-0.54)
t_{PTEF}	0.21 (0.05)	0.20 (0.06; 0.11-0.45)	0.16 (0.04)	0.15 (0.05; 0.12-0.26)	0.15 (0.01)	0.15 (0.02; 0.11-0.17)

[a] Lower t_{PTEF}/t_E and t_{PTEF} equal to a t_{PTEF}/t_E <0.25 and a t_{PTEF} <0.17 seconds (<25th percentile), respectively. t_{PTEF}/t_E: time to peak tidal expiratory flow to total expiratory time; t_{PTEF}: time to peak tidal expiratory flow

Supplementary Table S2. 1,2. Linear regression models of high TEWL, eczema, *FLG* mutations on the continuous t_{PTEF}/t_E (2.1) and t_{PTEF} (2.2), in the 899 three-month-old infants.

Characteristics	Crude β (95% CI)	*p* -Value	Adjusted β (95% CI)	*p* -Value [a]
		**Supplementary Table S2.1.** Continuous t_{PTEF}/t_E		
High TEWL [b]	0.00 (-0.01, 0.02)	0.689	-0.01 (-0.02, 0.01)	0.357
Eczema	0.00 (-0.01, 0.02)	0.559	0.00 (-0.02, 0.02)	0.861
FLG mutations	0.01 (-0.01, 0.03)	0.260	0.00 (-0.02, 0.03)	0.807
		**Supplementary Table S2.2.** Continuous t_{PTEF}		
High TEWL [b]	-0.01 (-0.01, 0.00)	0.205	-0.01 (-0.20, -0.00)	**0.029**
Eczema	-0.00 (-0.01, 0.01)	0.607	-0.01 (-0.02, 0.01)	0.373
FLG mutations	0.01 (-0.01, 0.02)	0.246	0.00 (-0.01, 0.02)	0.603

[a] Models adjusted for parental asthma, high parental education (≥ 4 years of university studies), nicotine exposure in pregnancy, sex, GA, weight at three months of age and the skin intervention. [b] High TEWL equal to a mean TEWL >8.83 $g/m^2/h$ (>75th percentile). TEWL: transepidermal waterloss; *FLG*: Filaggrin; t_{PTEF}/t_E: time to peak tidal expiratory flow to total expiratory time; t_{PTEF}: time to peak tidal expiratory flow.

Supplementary Table S3. 1,2. Interaction effects in logistic regression models of high TEWL, eczema, *FLG* mutations and the skin intervention on lower t_{PTEF}/t_E (3.1) and t_{PTEF} (3.2), in the 899 three-month-old infants.

Characteristics	Crude OR (95% CI)	*p* -Value	Adjusted OR (95% CI)	*p* -Value [(a)]
		**Supplementary Table S3.1.** Lower t_{PTEF}/t_E [(b)]		
High TEWL [(c)] × Skin intervention	0.39 (0.63, 1.39)	0.412	2.54 (0.52, 12.5)	0.250
Eczema × Skin intervention	0.93 (0.28, 3.08)	0.902	0.53 (0.08, 3.51)	0.530
FLG mutations × Skin intervention	0.48 (0.06, 3.58)	0.473	0.31 (0.02, 4.13)	0.378
		**Supplementary Table S3.2.** Lower t_{PTEF} [(b)]		
High TEWL [(c)] × Skin intervention	1.02 (0.65, 1.62)	0.924	0.67 (0.30, 1.50)	0.328
Eczema × Skin intervention	1.11 (0.61, 2.00)	0.734	1.33 (0.51, 3.47)	0.557
FLG mutations × Skin intervention	0.99 (0.46, 2.15)	0.990	0.70 (0.19, 2.55)	0.589

[(a)] Models adjusted for parental asthma, high parental education (≥ 4 years of university studies), nicotine exposure in pregnancy, sex, GA and weight at three months of age. [(b)] Lower t_{PTEF}/t_E and t_{PTEF} equal to a t_{PTEF}/t_E <0.25 and a t_{PTEF} <0.17 seconds (<25th percentile), respectively. [(c)] High TEWL equal to a mean TEWL >8.83 g/m²/h (>75th percentile). TEWL: transepidermal waterloss; *FLG*: Filaggrin; t_{PTEF}/t_E: time to peak tidal expiratory flow to total expiratory time; t_{PTEF}: time to peak tidal expiratory flow

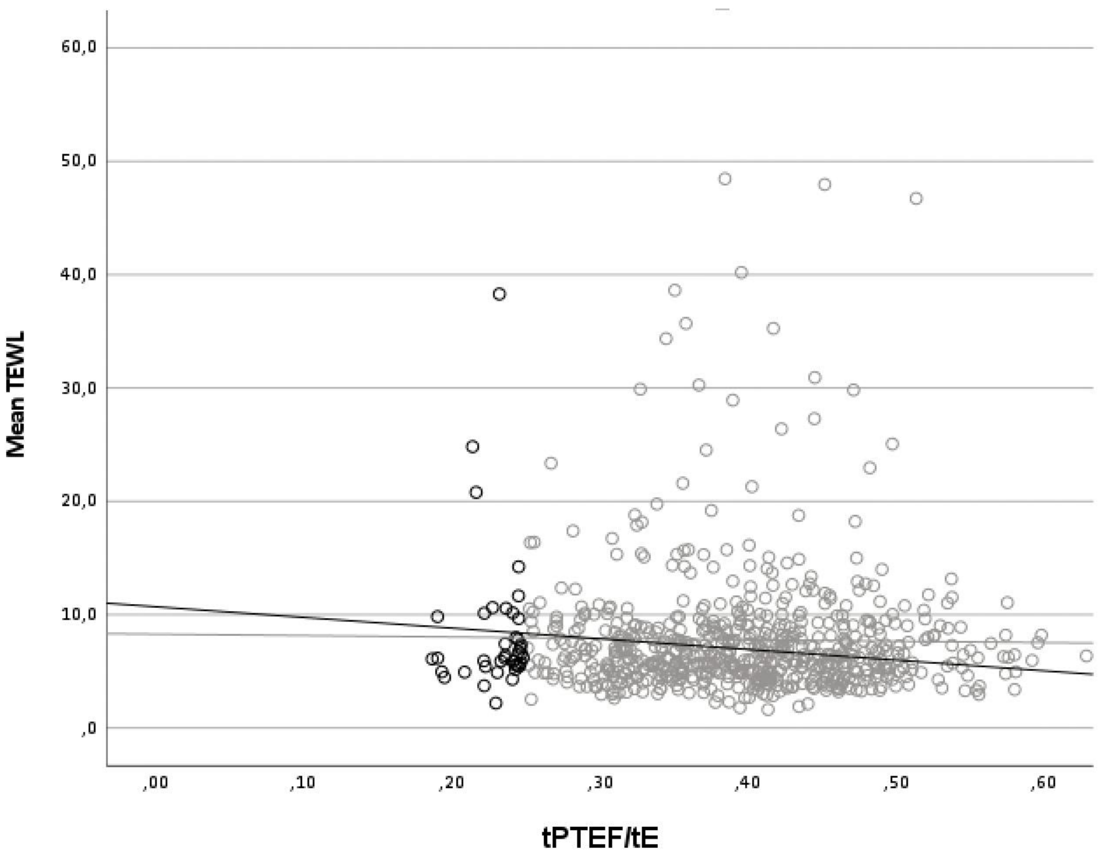

(a)

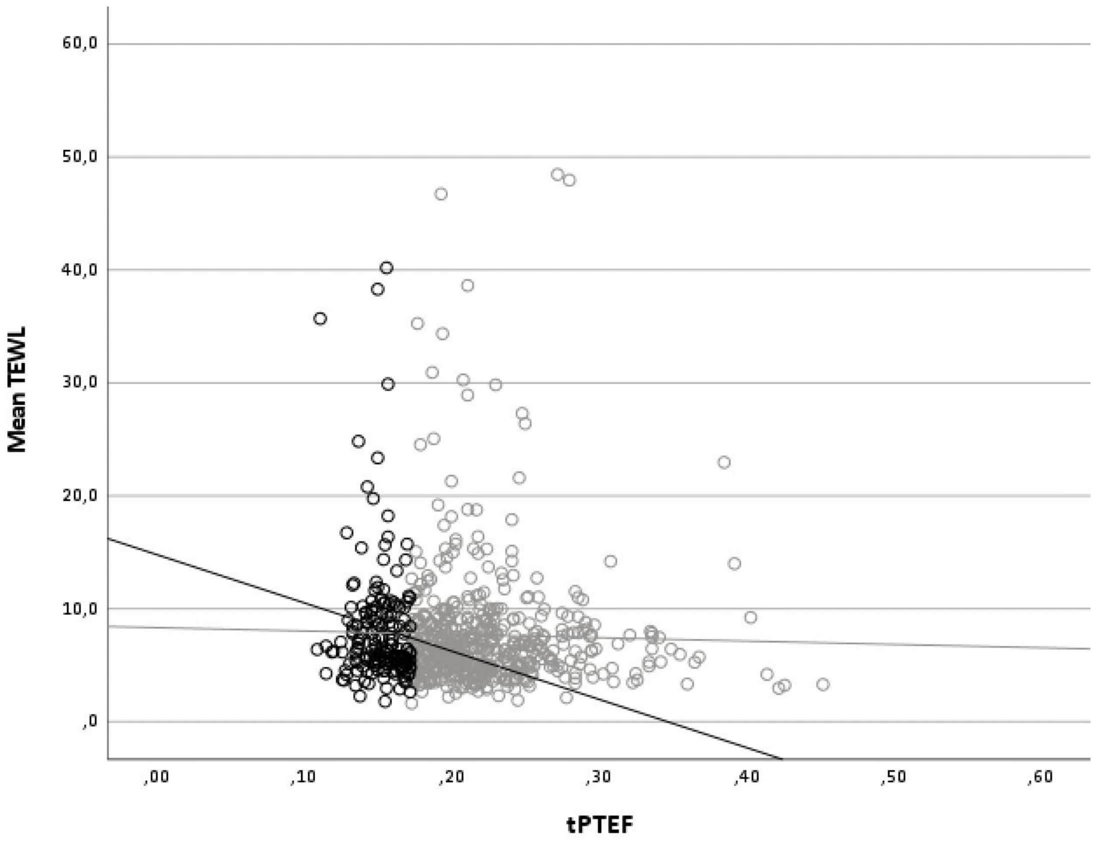

(**b**)

Supplementary Figure S1. (a,b). The distribution of the mean TEWL and the continuous t_{PTEF}/t_E (a) as well as t_{PTEF} (b) at three months of age. Black dots represent infants with lower t_{PTEF}/t_E and t_{PTEF} [(a)], respectively.

[(a)] Lower t_{PTEF}/t_E and t_{PTEF} equal to a t_{PTEF}/t_E <0.25 and a t_{PTEF} <0.17 seconds (<25th percentile), respectively
TEWL: transepidermal waterloss; t_{PTEF}/t_E: time to peak tidal expiratory flow to total expiratory time; t_{PTEF}: time to peak tidal expiratory flow
